# Major depressive disorders in young immigrants: A cohort study from primary healthcare settings in Sweden

**DOI:** 10.1177/14034948211019796

**Published:** 2021-06-14

**Authors:** Mehdi Osooli, Henrik Ohlsson, Jan Sundquist, Kristina Sundquist

**Affiliations:** 1 Center for Primary Health Care Research, Lund University, Sweden; 2 Department of Family Medicine and Community Health, Icahn School of Medicine at Mount Sinai, USA; 3 Department of Functional Pathology, Shimane University, Japan

**Keywords:** First-generation, immigrant, major depressive disorders, primary healthcare, registry, second-generation, Sweden

## Abstract

**Aims:**

Previous studies on major depressive disorder (MDD) among immigrants have reported mixed results. Using data from primary healthcare settings in Sweden, we compared the incidence of MDD among first- and second-generation immigrants aged 15–39 years with natives.

**Methods:**

This was a retrospective nationwide open cohort study. Eligible individuals were born 1965–1983, aged 15–39 years at baseline, and resided in Sweden for at least one year during the study period 2000–2015. We identified MDD cases through the Primary Care Registry (PCR). The follow-up for each individual started when they met the inclusion criteria and were registered in the PCR and ended at MDD diagnosis, death, emigration, moving to a county without PCR coverage, or the end of the study period, whichever came first. Results: The final sample included 1,341,676 natives and 785,860 immigrants. The MDD incidence rate per 1000 person-years ranged from 6.1 (95% confidence intervals: 6.1, 6.2) to 16.6 (95% confidence intervals: 16.2, 17.0) in native males and second-generation female immigrants with a foreign-born father, respectively. After adjusting for income, the MDD risk did not differ substantially between first-generation male and female immigrants and natives. However, male and female second-generation immigrants had a 16–29% higher adjusted risk of MDD than natives.

**Conclusions:**

This cohort study using primary healthcare data in Sweden, albeit incomplete, indicated that second-generation immigrants seem to be at a particularly high risk of MDDs. The underlying mechanisms need further investigation.

## Introduction

Millions of people have left their home countries due to war, political conflicts and economic hardship over the past decades. Based on United Nations estimates, the global immigrant population reached 272 million in 2019 [1]. Good mental health can help shape more successful integration in immigrant groups. However, many immigrants have experienced stressful and traumatizing situations that could affect both their own and their offspring’s mental health.

Previous research has shown that many first- and second-generation immigrants have an increased risk of developing mental disorders [2,3]. In Europe, young refugees and asylum-seekers had a reportedly higher risk of post-traumatic stress disorder (PTSD) [4,5], emotional and behavioral disorders and anxiety disorders. A recent global systematic review found a higher risk of mood disorders among first- and second-generation immigrants than native populations [6].

Major depressive disorder (MDD) is a common mood disorder and a leading cause of lost productivity and poor health-related quality of life. Between 1990 and 2017, the global age-adjusted incidence rates of MDD increased in most countries [7]. In 2017, MDD accounted for an estimated 2.5% (95% confidence interval (CI): 1.8, 3.2) of the lost disability-adjusted life years (DALY) among those aged 15–49 years [8]. Persons living with MDD also have an increased risk of suicide [9], alcohol dependence and drug addiction [10]. Genetic vulnerability (e.g. family history of MDD) and environmental factors (e.g. childhood adverse experiences) and their interplay may increase the risk of MDD [11].

Systematic review studies on rates of MDD among immigrants have reported heterogeneous and mixed results [4,6,12,13]. Insufficient sample sizes, use of different definitions of immigrants and suboptimal evaluation of MDD may in part account for the observed heterogeneities. Also, in past studies, more severe forms of MDD, that is those requiring hospital care, have received more attention than cases where hospitalization is not required, although Sundquist et al. have indicated that nearly 80% of MDD cases are managed in primary care settings [14]. Data on the incidence of MDD among immigrant populations based on primary healthcare diagnoses are, however, limited. Representative samples and longitudinal data sources are needed to estimate the incidence of MDD in immigrant populations.

Over the past few decades, Sweden has received a large number of immigrants including refugees/asylum seekers and non-refugees (e.g. students, labour immigrants and immigrants coming to Sweden due to family ties). A large number of refugees in Sweden originate from the Middle East and Africa, while many non-refugee immigrants are from European countries. By 2018, approximately 2,540,000 Swedish residents (around 25% of the population) had an immigrant background including first-generation immigrants (born abroad) and second-generation immigrants (born in Sweden with one or two foreign-born parents).

All residents of Sweden, including immigrants and non-immigrants, have access to affordable healthcare services that are universal. Primary care services are available within a short distance in most neighborhoods and have a high coverage in the country. The majority of the Swedish population will therefore seek healthcare through public services. Sweden also has a tradition of nationwide individual-level data collection for administrative purposes. The availability of longitudinal databases and the large number of immigrants creates a unique opportunity to study immigrant mental health with a high statistical power and at a reasonably low cost. A Swedish Primary Care Register (PCR), covering all primary healthcare visits for approximately 87% of the Swedish population, has recently become available for research purposes. To the best of our knowledge, this study is the first to use large-scale primary healthcare data to estimate incidence rates and risks of MDD among first- and second-generation immigrants compared to natives aged 15–39 years.

## Methods

In this retrospective open cohort study, we used individual-level data from several nationwide registers in Sweden. The individual-level data across registers were linked using a unique personal identification number, which, to preserve confidentiality, was replaced with a serial number by Statistics Sweden. The study population consisted of 2,127,536 individuals born 1 January 1965 to 31 December 1983, who resided in Sweden for at least one year between 1 January 2000 and 31 December 2015. This study was part of a larger project which received ethical approval from the Regional Ethical Review Board in Lund, Sweden (Ethics approval No: 2012/795). The latest amendment was approved by the Swedish Ethical Review Authority (Ethics approval No: 2019-01588).

### Data sources and variables

We used individual-level data from the Register of the Total Population (RTB), the Multi-Generation Register, the Cause of Death Register, and the Migration Register to identify eligible individuals. Furthermore, we used the Longitudinal Integration Database for Health Insurance and Labor Market Studies (LISA) as the source of data on income. Statistics Sweden (SCB) and The National Board of Health and Welfare (*Socialstyrelsen*) provided us with most of the data for this analysis. For the outcome MDD, clinical diagnoses were obtained from the PCR. The PCR was created from regional data sources from a majority of Swedish counties.

#### Immigrant background

We defined natives as individuals with both parents born in Sweden. The immigrant group were assigned to first- or second-generation immigrants. Individuals in the first-generation immigrant group were born abroad with both parents born abroad. Second-generation immigrants were born in Sweden and were divided into three subgroups based on parental country of birth: (a) both parents foreign-born; (b) Swedish-born father and foreign-born mother; and (c) Swedish-born mother and foreign-born father. The individuals were distributed as follows: 1,341,676 natives (63.0%), 550,304 (25.9%) first-generation immigrants (with two foreign-born parents), 82,550 (3.9%) second-generation immigrants with two foreign-born parents, and 153,006 (7.2%) second-generation im-migrants with one foreign-born parent.

#### Income

We obtained individual disposable income from the Swedish Tax Agency for the individuals who were 18 years or older at baseline. This data has nationwide population coverage and is highly accurate. For individuals younger than 18 years, we used maternal income at baseline. Disposable income was standardized per year to make the values comparable over time. We categorized the income variable into quartiles (low, mid-low, mid-high and high income). Only 11,117 individuals had a missing income. These few individuals were considered very likely to have no income at all. Therefore, we assigned them to the low-income quartile.

#### MDD diagnosis

**Figure 1.**
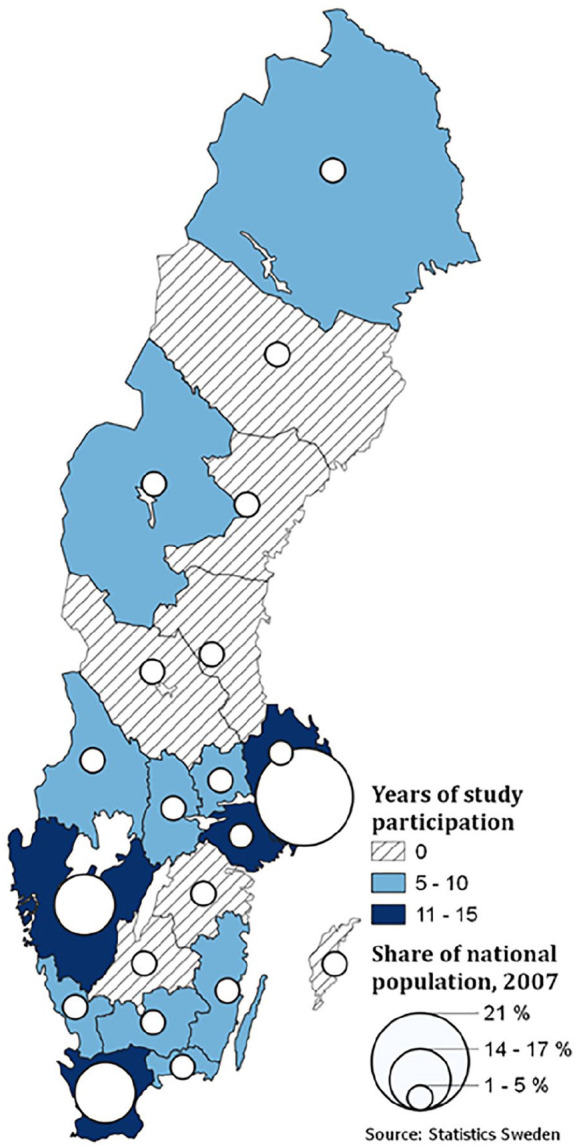
The number of years with Primary Care Registry (PCR) coverage during follow-up and the distribution of the Swedish population (in 2007) across provinces in Sweden.

We identified individuals with MDD through the PCR. The PCR includes individual-level information on clinical diagnoses at visits to primary healthcare centers in Sweden. The PCR covered the majority of Swedish counties during the follow-up. The digitalization of patient records at primary healthcare centers started, however, in different years across counties. Figure 1 presents the population density in 2007 (mid-follow-up time) and the number of years each county contributed with data during the follow-up period. We used the 10th revision of the International Classification of Diseases (ICD-10), codes F32 and F33, to identify individuals with MDD.

### Statistical analysis

Baseline was defined as the year the individual was registered in a county that was covered in the PCR. The follow-up ended at the time of an MDD registration, death, emigration, moved to a county without coverage in the PCR or the end of the study period (31 December 2015), whichever came first. Age-adjusted incidence rates (IR) of MDD were estimated per 1000 person-years and by sex and immigrant group. Incidence was defined as first registration of MDD during the study period. Incidence rate ratios (IRRs) (using Swedish natives with two Swedish-born parents as the reference group) were calculated for males and females separately. Model A included adjustment for birth year and Model B also controlled for income. For all estimates, 95% CIs were used. We also calculated period prevalence of MDD for the immigrant and non-immigrant groups.

## Results

Table I shows the characteristics of the study population by the covariates and the outcome. First-generation immigrants had the highest percentage of individuals who moved to a region without PCR coverage. The follow-up period ranged from 5.9 (first-generation immigrants) to 9.3 years (second-generation immigrants with two-foreign-born parents). Among the natives and the immigrant groups, 117,501 and 61,964 individuals had an MDD diagnosis, respectively.

**Table I.** Characteristics of individuals with a native or an immigrant background, aged 15–39 years, Sweden.

	**Native Swedish**	**First-generation**	**Second-generation**		
		Two foreign-born parents	Two foreign-born parents	Foreign-born mother^a^	Foreign-born father^a^
**Sex** (*n* (%))					
Male	688,815 (51.3)	282,726 (51.4)	42,611 (51.6)	36,696 (51,5)	41,860 (51.2)
Females	652,861 (48.7)	267,578 (48.6)	39,939 (48.4)	34,596 (48.5)	39,854 (48.8)
**Age at baseline** (*n* (%))					
15–19	125,102 (9.3)	19,087 (3.5)	9263 (11.2)	7490 (10.5)	8367 (10.2)
20–24	278,564 (20.8)	74,943 (13.6)	21,807 (26.4)	16,192 (22.7)	18,373 (22.9)
25–29	365,259 (27.2)	163,744 (29.8)	22,547 (27.3)	18,412 (25.8)	22,160 (27.1)
30–39	572,751 (42.7)	292,530 (53.2)	28,933 (35.1)	29,198 (41.0)	32,450 (39.0)
**Age at end of follow up** (*n* (%))					
15–19	719 (0.1)	382 (0.1)	72 (0.1)	66 (0.1)	55 (0.1)
20–24	13,558 (1.0)	8186 (1.5)	1337 (1.6)	976 (1.4)	1199 (1.5)
25–29	44,089( 3.3)	37,625 (6.8)	4149 (5.0)	3214 (4.5)	3604 (4.4)
30–40	1,283,310 (95.7)	504,111 (91.6)	76,992 (93.3)	67,063 (94.0)	76,856 (94.1)
**Income**^b^ (*n* (%))					
Quartile 1 (lowest)	185,517 (13.8)	302,821 (55.0)	15,988 (19.4)	11,158 (15.7)	13,597 (16.6)
Quartile 2	344,772 (25.7)	112,951 (20.5)	27,842 (33.7)	19,904 (27.9)	23,728 (29.0)
Quartile 3	394,679 (29.4)	64,330 (11.7)	23,625 (28.6)	22,020 (30.9)	24,526 (30.0)
Quartile 4 (highest)	416,224 (31.0)	59,681 (10.9)	15,057 (18.2)	18,173 (25.5)	19,826 (24.3)
Missing	484 (0.1)	10,521 (1.9)	38 (0.1)	37 (0.1)	37 (0.1)
**Reason for ending follow-up** (*n* (%))					
Died	10,502 (0.8)	2548 (0.5)	988 (1.2)	726 (1.0)	824 (1.0)
Emigrated	1717 (0.1)	15,626 (2.8)	177 (0.2)	126 (0.2)	151 (0.2)
Study ended/turned 40 years	893,554 (66.6)	298,438 (54.2)	52,067 (63.1)	46,727 (65.5)	52,716 (64.5)
Moved to a province without PCR coverage	319,508 (23.8)	198,210 (36.0)	19,977 (24.2)	16,352 (22.9)	18,830 (23.0)
**Had an MDD diagnosis** (*n* (%))					
Yes	117,501 (8.8)	35,737 (6.5)	9478 (11.5)	7442 (10.4)	9307 (11.4)
No	1,224,175 (91.2)	514,567 (93.5)	73,072 (88.5)	63,850 (89.6)	72,407 (88.6)
**Person-years of follow up** (mean (SD))	8.8 (4.2)	5.9 (4.0)	9.3 (4.2)	8.9 (4.2)	9.0 (4.2)

a The other parent was Swedish-born.

b We used the maternal income and own income at baseline for individuals < 18 and ⩾ 18 years, respectively.

PCR, Primary Care Registry; MDD, major depressive disorders.

Table II shows the prevalence and incidence rates of MDD in the different subgroups. The prevalence in males varied between 4.3 (95% CI: 4.2–4.4) in first-generation immigrants and 7.8 in two of the second-generation immigrant subgroups. The prevalence in females was approximately twice as high in each subgroup and varied between 8.8 (95% CI: 8.7–8.9) in first-generation female immigrants and 16.6 (95% CI: 16.2–17.0) in second-generation female immigrants with a foreign-born father. The age-adjusted incidence rates in males varied between 6.1 (95% CI: 6.1–6.2) in natives and 8.2 (95% CI: 7.9–8.5) in second-generation immigrants with a foreign-born father. The corresponding incidence rates in females was approximately twice as high in each subgroup.

**Table II.** Prevalence and age-adjusted incidence rates (IR) per 1000 person-years of major depressive disorders (MDDs) among first- and second-generation immigrants and natives, aged 15–39 years, Sweden.

	Male				Female			
	At risk (*n*)	Cases (*n*)	Prevalence^a^ (95 % CI)	IR (95 % CI)	At risk (*n*)	Cases (*n*)	Prevalence^a^ (95 % CI)	IR (95 % CI)
**Native Swedish**	688,815	39,571	5.7 (5.7, 5.8)	6.1 (6.1, 6.2)	652,861	77,930	11.9 (11.9, 12.0)	13.4 (13.2, 13.3)
**First-generation immigrants**								
Two foreign-born parents	282,726	12,137	4.3 (4.2, 4.4)	7.1 (6.9, 7.2)	267,578	23,600	8.8 (8.7, 8.9)	14.1 (13.9, 14.2)
**Second-generation immigrants**								
Two foreign-born parents	42,611	3327	7.8 (7.5, 8.1)	7.8 (7.5, 8.1)	39,939	6151	15.4 (15.0, 15.7)	16.4 (16.0, 16.8)
Foreign-born mother^b^	36,696	2,577	7.0 (6.8, 7.3)	7.4 (7.2, 7.7)	34,596	4865	14.1 (13.7, 14.4)	15.7 (15.2, 16.1)
Foreign-born father^b^	41,860	3,276	7.8 (7.6, 8.1)	8.2 (7.9, 8.5)	39,854	6031	15.1 (14. 8, 15.5)	16.6 (16.2, 17.0)

a Estimated based on the entire follow-up period 2000-2015.

b The other parent was born in Sweden.

CI, confidence interval: IR, incidence rate; ICD codes for MDDs, F32, F33.

Table III shows the IRRs of MDD comparing the male and female immigrant subgroups with the native reference group after adjusting for birth year in Model A and, in Model B, additional adjustment for income. In the Model A for males, immigrants had higher risks of MDD compared with natives with IRRs ranging between 1.15 (95% CI: 1.13, 1.17) and 1.34 (95% CI: 1.29, 1.39) in first-generation immigrants and second-generation immigrants with a foreign-born father, respectively. After adjustment for income, the risks decreased slightly but remained significant in all subgroups. In Model A for females, immigrants also had higher risks of MDD compared with natives with IRRs ranging between 1.06 (95% CI: 1.04, 1.08) and 1.25 (95% CI: 1.22, 1.28) in first-generation immigrants and second-generation immigrants with a foreign-born father, respectively, that is the same subgroups as in males. After additional adjustment for income, the risks decreased slightly and no longer remained significant in first-generation female immigrants.

**Table III.** Incidence rate ratios (IRRs) of major depressive disorders (MDDs) among male and female individuals aged 15–39 years with an immigrant background compared to natives in Sweden.

	Male		Female	
	Model A IRR (95% CI)	Model B IRR (95% CI)	Model A IRR (95% CI)	Model B IRR (95% CI)
First generation				
Two foreign-born parents	1.15 (1.13, 1.17)	1.06 (1.03, 1.08)	1.06 (1.04, 1.08)	1.01 (0.99, 1.03)
Second generation				
Two foreign-born parents	1.27 (1.23, 1.32)	1.19 (1.15, 1.24)	1.23 (1.20, 1.27)	1.19 (1.16, 1.22)
Foreign-born mother^a^	1.21 (1.16, 1.26)	1.18 (1.14, 1.23)	1.18 (1.14, 1.21)	1.16 (1.13, 1.20)
Foreign-born father^a^	1.34 (1.29, 1.39)	1.29 (1.25, 1.34)	1.25 (1.22, 1.28)	1.23 (1.19, 1.26)

a The other parent was born in Sweden.

CI, confidence interval; ICD code for MDDs, F32, F33; Model A includes birth year; Model B also controls for income.

## Discussion

We conducted a retrospective cohort study based on primary healthcare diagnoses among 2 million individuals aged 15–39 years in Sweden and compared the incidence rates of MDD between natives and first- and second-generation immigrants. After controlling for the effects of income on MDD, all male and female second-generation immigrant groups had a 16–29% higher risk of MDD than the native reference group. However, the MDD risk in male and female first-generation immigrants did not differ substantially from the reference group, after adjusting for income.

Previous research on MDD risk among first-generation immigrants is heterogeneous [13]. The first-generation immigrants in our study did not have a substantially different MDD risk from natives. This finding contrasted with the results of previous studies from other countries [15–17]. In Canada, a systematic review indicated a lower prevalence of mood disorders among first-generation immigrants than natives [15]. The review also highlighted a lack of longitudinal studies on mood disorders among immigrants in Canada. In Finland, a register-based study found lower rates of any psychiatric disorder among first-generation immigrants than natives [17]. A meta-analysis that included 25 surveys performed in Western countries indicated a lower prevalence of major depression in first-generation immigrants compared to the general populations [4].

However, some research has suggested an elevated risk of mood disorders, such as depression, among first-generation immigrants [4,18]. A systematic review by Mindlis et al. indicated an approximately 25% higher risk of mood disorders in first-generation immigrants than natives [6]. A large multicenter survey, initiated by the World Health Organization (WHO) in France, showed a higher likelihood of mood disorders among first-generation immigrants [16]. A systematic review showed a two-fold prevalence of depression among first-generation refugees than labor immigrants [18]. Other studies were in line with ours showing no substantially different depression risk compared with natives. For example, in the Netherlands, there were no differences between female immigrants from Turkey and Morocco and native controls in the risk of hospitalization for depressive disorders [19]. Foo et al. conducted a systematic review on first-generation immigrants and found a 10% lower but statistically non-significant odds of depression in this group compared with natives [12].

Limited data exist on the risk of MDD and other mood disorders among second-generation immigrants [6]. The second-generation immigrant subgroups in our study had an approximately 15–30% higher risk of MDD than natives. This finding is in partial agreement with previous research. Lau et al. showed, in a nationally representative survey, that the second-generation Asian American women in the USA had higher rates of depression than their first-generation counterparts [20]. A register-based study from Sweden showed a higher risk of affective disorders in second-generation Finns [21]. However, other second-generation immigrant subgroups in the study did not have higher risks of affective disorders than natives. In a systematic review from 2017, a slightly higher risk of mood disorders among second-generation immigrants compared with natives was found but it did not reach statistical significance [6]. In our study, the risk of MDD among second-generation immigrants did not vary substantially in the different subgroups. Our results partially supported the findings of a study from Denmark [22]. Cantor-Grae et al. used register-based data on hospital admissions for any psychiatric disorder in Denmark and found that second-generation immigrants with one foreign-born parent had a higher risk than natives [22]. However, the second-generation with two-foreign-born parents had a lower risk of hospital admission for any psychiatric disorder than natives. In contrast to our findings, the results of a nationally representative survey in the USA did not find a higher risk for depressive disorders among second-generation young adult immigrants than natives [23]. Another study from the USA found varying risks of mood disorders across second-generation immigrants from different ethnic groups [24].

However, it is important to keep in mind that the existing heterogeneities between different studies may be explained by differences in reason for immigration. For example, many refugees and asylum seekers may experience larger difficulties in learning the new language and securing an employment whereas labour immigrants may have better possibilities to achieve a successful integration in the host country. Heterogeneities in previous studies may also be related to different ways to measure the outcome. Our study was based on clinical diagnoses from primary healthcare settings, whereas most previous studies were based on surveys or hospital data.

MDD is a challenging psychiatric diagnosis with key symptoms ranging from a somewhat depressed mood to suicidal behavior and completed suicide [25]. Genetic studies have suggested a moderate heritability for MDD [26] whereas psychosocial risk factors seem to play significant roles in the development of the condition during the lifespan [25]. Immigrants experience substantial stress before, during and after migration. Although this study did not investigate causal mechanisms behind the differential risks of MDD among the immigrant groups, there are several potential explanations where only a few can be elaborated upon here. Firstly, economic and employment challenges are among the main determinants of mental health in immigrants. However, we controlled for income in our estimates and the results remained almost unchanged. It is possible that other work-related factors, including employment stability and job satisfaction, represent potential determinants of immigrants’ mental health. Immigrants may be at a higher risk of losing their jobs than natives. Poorer social networks, employment opportunities and economic challenges might hit the immigrants harder than the natives. In addition, later generations of immigrants may experience an even more pronounced psychosocial stress concerning discrimination, which puts them at higher risks of mental health issues [20]. Acculturative stress may also increase the risk of depressive disorders among immigrant populations.

It is possible that the risk of MDD among the first-generation immigrants in our study is underestimated due to a lower healthcare service utilization [24]. Castaneda et al. investigated potential disparities in immigrants’ uptake of mental health and rehabilitation services using combined survey- and register-based data in Finland [27]. According to their results, among those with affective symptoms, immigrants had a lower uptake of mental health and rehabilitation services than native Finns. However, in a register-based study from Denmark, male and female immigrants had a higher risk of a first-time contact for mental disorders than natives [28] and all immigrant subgroups, except those originating from Asia and Sub-Saharan Africa, had a higher likelihood of seeking psychiatric services for affective disorders than natives.

As young second-generation immigrants tend to be better educated than their parents, they may be more likely to use psychiatric services. Another study showed that the longer time a first-generation immigrant had lived in the country, the more likely they were to use psychiatric services [29]. First-generation immigrants may also perceive depression differently than natives. A qualitative study from France found four main reasons why the immigrants did not seek help for their depression at primary health clinics: the reluctance to be treated (pharmacological treatment and/or psychotherapy), the preference to see a psychiatrist directly, providential healing and not believing that treatment is necessary. First-generation immigrants were less likely to talk to doctors about their depressive symptoms despite the majority of them appreciating more support. The immigrants may also be more likely to endorse self-help strategies to deal with their mental health issues. Perceived stigma towards psychiatric diagnoses is another barrier to service utilization [30].

Our study had some limitations. First, we only controlled for income at baseline. The impact of variations in the income during the follow-up was not accounted for. Second, during the follow-up, some individuals moved to areas without coverage in the PCR. First-generation immigrants were more likely to move to regions without PCR coverage. This may have underestimated the risk of MDDs in the first-generation immigrants in our study. However, considering that the differences in mobility between the subgroups were not large, it should not have changed the main conclusions of this study to a large extent. Third, we don’t have any estimates on the validity of MDD diagnoses in primary healthcare settings. The MDD diagnoses in primary healthcare settings are, in most cases, given by physicians and, in some cases, by certified psychologists. Medical diagnoses in Sweden have, in general, high validity [31] and it could be assumed that this is the case for primary healthcare diagnoses as well. Finally, our study was explorative and we did not investigate the potential impact of differences in service utilization, medical treatment and psychotherapy or risk factors of poor mental health that may differ between different immigrant groups (i.e. refugees v. labour immigrants, European v. non-European immigrants) and natives. Our study also had many strengths. The large sample size and the use of unique almost nationwide primary care data increase the generalizability of our findings. The data from the majority of primary healthcare centers in Sweden was available over the 16 years of this study. In addition, more than one assessment is needed to identify an MDD diagnosis. Therefore, the data on clinical MDD diagnoses in our study may be more representative compared to surveys based on self-report at only one assessment. However, we did not have access to detailed information on diagnostic procedures and evaluations.

## Conclusions

We investigated the risk of MDD among first- and second-generation immigrant subgroups based on almost nationwide clinical data from primary healthcare centers in Sweden. The first-generation immigrants had an MDD risk that was comparable with the one in natives. However, the MDD risk among all second-generation immigrant subgroups was higher than in natives. These results urge for further investigations to find potential explanations behind our findings, such as a lower healthcare utilization in the first generation and/or a poor integration in the second generation.

## References

[1] United Nations Department of Economic and Social Affairs. International migrants numbered 272 million in 2019, continuing an upward trend in all major world regions: Population Facts, United Nations, https://www.un.org/en/development/desa/population/migration/data/estimates2/estimates19.asp (2019).

[2] Cano MA Schwartz SJ Castillo LG , et al. Depressive symptoms and externalizing behaviors among Hispanic immigrant adolescents: examining longitudinal effects of cultural stress. J Adolesc 2015;42:31–39. 25899132 10.1016/j.adolescence.2015.03.017 PMC4464969

[3] Norredam M Nellums L Nielsen RS , et al. Incidence of psychiatric disorders among accompanied and unaccompanied asylum-seeking children in Denmark: a nation-wide register-based cohort study. Eur child Adolesc Psychiatry 2018;27:439–446. 29488029 10.1007/s00787-018-1122-3

[4] Fazel M Wheeler J Danesh J. Prevalence of serious mental disorder in 7000 refugees resettled in western countries: a systematic review. Lancet 2005;365:1309–1314. 15823380 10.1016/S0140-6736(05)61027-6

[5] Mood C Jonsson JO Låftman SB. Immigrant integration and youth mental health in four European countries. Eur Sociol Rev 2016;32:716–729.

[6] Mindlis I Boffetta P. Mood disorders in first- and second-generation immigrants: systematic review and meta-analysis. Br J Psychiatry 2017;210:182–189. 28069564 10.1192/bjp.bp.116.181107

[7] Liu Q He H Yang J , et al. Changes in the global burden of depression from 1990 to 2017: findings from the Global Burden of Disease study. J Psychiatr Res 2020; 126:134–140. 31439359 10.1016/j.jpsychires.2019.08.002

[8] Network. GBoDC. Global Burden of Disease Study 2017 (GBD 2017), http://vizhub.healthdata.org/gbd-compare/ (2018, accessed 2 December 2018).

[9] Li Z Page A Martin G , et al. Attributable risk of psychiatric and socio-economic factors for suicide from individual-level, population-based studies: a systematic review. Soc Sci Med 2011;72:608–616. 21211874 10.1016/j.socscimed.2010.11.008

[10] Conner KR Pinquart M Gamble SA. Meta-analysis of depression and substance use among individuals with alcohol use disorders. J Subst Abuse Treat 2009;37:127–137. 19150207 10.1016/j.jsat.2008.11.007 PMC4864601

[11] Kwong ASF Lopez-Lopez JA Hammerton G , et al. Genetic and environmental risk factors associated with trajectories of depression symptoms from adolescence to young adulthood. JAMA Netw Open 2019;2:e196587. 31251383 10.1001/jamanetworkopen.2019.6587 PMC6604106

[12] Foo SQ Tam WW Ho CS , et al. Prevalence of depression among migrants: a systematic review and meta-analysis. Int J Environ Res Public Health 2018;15:1986. 30213071 10.3390/ijerph15091986 PMC6163821

[13] Close C Kouvonen A Bosqui T , et al. The mental health and wellbeing of first generation migrants: a systematic-narrative review of reviews. Global Health 2016; 12:47. 27558472 10.1186/s12992-016-0187-3 PMC4997738

[14] Sundquist J Ohlsson H Sundquist K , et al. Common adult psychiatric disorders in Swedish primary care where most mental health patients are treated. BMC Psychiatry 2017;17:235. 28666429 10.1186/s12888-017-1381-4 PMC5493066

[15] Edwards J Hu M Thind A , et al. Gaps in understanding of the epidemiology of mood and anxiety disorders among migrant groups in Canada: a systematic review. Can J Psychiatry 2019;64:595–606. 31129987 10.1177/0706743719839313 PMC6699028

[16] Pignon B Geoffroy PA Thomas P , et al. Prevalence and clinical severity of mood disorders among first-, second- and third-generation migrants. J Affect Disord 2017;210:174–180. 28049102 10.1016/j.jad.2016.12.039

[17] Markkula N Lehti V Gissler M , et al. Incidence and prevalence of mental disorders among immigrants and native Finns: a register-based study. Soc Psychiatry Psychiatr Epidemiol 2017;52:1523–1540. 28856385 10.1007/s00127-017-1432-7

[18] Lindert J Ehrenstein OS Priebe S , et al. Depression and anxiety in labor migrants and refugees—a systematic review and meta-analysis. Soc Sci Med 2009;69:246–257. 19539414 10.1016/j.socscimed.2009.04.032

[19] Selten JP van Os J Nolen WA. First admissions for mood disorders in immigrants to the Netherlands. Soc Psychiatry Psychiatr Epidemiol 2003;38:547–550. 14564382 10.1007/s00127-003-0673-9

[20] Lau AS Tsai W Shih J , et al. The immigrant paradox among Asian American women: are disparities in the burden of depression and anxiety paradoxical or explicable? J Consult Clin Psychol 2013;81:901–911. 23477477 10.1037/a0032105 PMC3835700

[21] Saraiva Leao T Sundquist J Johansson LM , et al. Incidence of mental disorders in second-generation immigrants in sweden: a four-year cohort study. Ethn Health 2005;10:243–256. 16087456 10.1080/13557850500096878

[22] Cantor-Graae E Pedersen CB. Full spectrum of psychiatric disorders related to foreign migration: a Danish population-based cohort study. JAMA Psychiatry 2013;70:427–435. 23446644 10.1001/jamapsychiatry.2013.441

[23] Hao L Woo HS. Distinct trajectories in the transition to adulthood: are children of immigrants advantaged? Child Dev 2012;83:1623–1639. 22966927 10.1111/j.1467-8624.2012.01798.x PMC4479264

[24] Georgiades K Paksarian D Rudolph KE , et al. Prevalence of mental disorder and service use by immigrant generation and race/ethnicity among US adolescents. J Am Acad Child Adolesc Psychiatry 2018;57:280–287.e2. 29588054 10.1016/j.jaac.2018.01.020

[25] Malhi GS Mann JJ. Depression. The Lancet 2018;392:2299–2312. 10.1016/S0140-6736(18)31948-2 30396512

[26] Flint J Kendler KS. The genetics of major depression. Neuron 2014;81:484–503. 24507187 10.1016/j.neuron.2014.01.027 PMC3919201

[27] Castaneda AE Çilenti K Rask S , et al. Migrants are underrepresented in mental health and rehabilitation services—survey and register-based findings of Russian, Somali, and Kurdish Origin Adults in Finland. Int J Environ Res Public Health 2020;17:6223. 32867157 10.3390/ijerph17176223 PMC7504052

[28] Norredam M Garcia-Lopez A Keiding N , et al. Risk of mental disorders in refugees and native Danes: a register-based retrospective cohort study. Soc Psychiatry Psychiatr Epidemiol 2009;44:1023–1029. 19294322 10.1007/s00127-009-0024-6

[29] Manhica H Almquist Y Rostila M , et al. The use of psychiatric services by young adults who came to Sweden as teenage refugees: a national cohort study. Epidemiol Psychiatr Sci 2017;26:526–534. 27353562 10.1017/S2045796016000445 PMC6999002

[30] Szaflarski M Cubbins LA Bauldry S , et al. Major depressive disorder and dysthymia at the intersection of nativity and racial-ethnic origins. J Immigr Minor Health 2016;18:749–763. 26438660 10.1007/s10903-015-0293-y PMC4821814

[31] Ludvigsson JF Andersson E Ekbom A , et al. External review and validation of the Swedish national inpatient register. BMC Public Health 2011;11:450. 21658213 10.1186/1471-2458-11-450 PMC3142234

